# Double-Edged Sword of Tumour Suppressor Genes in Schizophrenia

**DOI:** 10.3389/fnmol.2019.00001

**Published:** 2019-02-12

**Authors:** Chuanjun Zhuo, Dawei Wang, Chunhua Zhou, Ce Chen, Jie Li, Hongjun Tian, Shen Li, Feng Ji, Chuanxin Liu, Min Chen, Li Zhang

**Affiliations:** ^1^Genetics Laboratory, Department of Neuroimaging, Department of Psychiatry, Nankai University Affiliated Anding Hospital, Tianjin Anding Hospital, Tianjin, China; ^2^Psychiatric Genetic Laboratory, Department of Psychiatry, Jining Medical University, Jining, China; ^3^Department of Psychiatric Genetics, Tianjin Medical University, Tianjin, China; ^4^Department of Psychiatry, Wenzhou Seventh People’s Hospital, Wenzhou, China; ^5^Department of Neuroimaging Laboratory, Qilu Hospital of Shandong University, Jinan, China; ^6^Department of Pharmacy, The First Hospital of Hebei Medical University, Shijiazhuang, China; ^7^GHM Institute of CNS Regeneration, Jinan University, Guangzhou, China

**Keywords:** schizophrenia risk gene, tumour suppressor gene, neurodevelopment, Wnt pathway, molecular targeting drugs

## Abstract

Schizophrenia (SCZ) is a common psychiatric disorder with polygenetic pathogenesis. Among the many identified candidate genes and loci, the group of tumour suppressor genes has drawn our interest. In this mini-review article, we describe evidence of a correlation between major tumour suppressor genes and SCZ development. Genetic mutations ranging from single nucleotide polymorphisms to large structural alterations have been found in tumour-related genes in patients with SCZ. Epigenetic mechanisms, including DNA methylation/acetylation and microRNA regulation of tumour suppressor genes, have also been implicated in SCZ. Beyond genetic correlations, we hope to establish causal relationships between tumour suppressor gene function and SCZ risk. Accumulating evidence shows that tumour suppressor genes may mediate cell survival and neural development, both of which contribute to SCZ aetiology. Moreover, converging intracellular signalling pathways indicate a role of tumour suppressor genes in SCZ pathogenesis. Tumour suppressor gene function may mediate a direct link between neural development and function and psychiatric disorders, including SCZ. A deeper understanding of how neural cell development is affected by tumour suppressors may lead to improved anti-psychotic drugs.

## Genetic Factors of Schizophrenia

Schizophrenia (SCZ) has a worldwide prevalence of approximately 1% (Leucht et al., 2007). The clinical symptoms of SCZ include delusions, hallucinations, social deficits, and emotional and cognitive impairments (Sass and Parnas, 2003). SCZ has become a major health burden as it affects patients’ well-being as well as the welfare of their families and society at large (Millier et al., 2014). The disease typically has an early onset around adolescence (Häfner et al., 1993), raising the possibility of genetic factors contributing to its pathogenesis. In addition, classical twin studies of SCZ have revealed a high heritability (Sullivan et al., 2003). However, early efforts to identify candidate SCZ genes by linkage analysis did not produce consistent results (Shi et al., 2008), suggesting that SCZ aetiology could not be attributed to a single gene mutation. We now understand that genetic contributions to SCZ are inherently complex and include structural variations and single-nucleotide variations (Chen et al., 2015). Modern genetic techniques, especially genome-wide association studies and next-generation DNA sequencing approaches, have significantly accelerated the identification of SCZ risk genes and loci. Perhaps unsurprisingly, these studies, using data from very large patient populations, have found that multiple gene variants are significantly associated with SCZ, further supporting the polygenic and heterogeneous nature of SCZ aetiology.

At present, the principal candidate loci for SCZ include the Disrupted in SCZ-1 gene, which regulates neurodevelopment (Miyoshi et al., 2003), and the major histocompatibility complex genes (Purcell et al., 2009), suggesting immune system involvement in SCZ pathology. Other major risk genes include those encoding transcription factors and synaptic proteins such as voltage-gated calcium channels (Schizophrenia Working Group of the Psychiatric Genomics Consortium, 2014). In addition, a group of tumour suppressor genes have also been associated with SCZ (Catts and Catts, 2000). Their possible involvement is bolstered by a recently published transcriptomic meta-analysis that demonstrated an inverse relationship between psychiatric disorders and cancer risk, and identified three major signalling cascades including the p53, Wnt, and peptidylprolyl cis/trans isomerase NIMA-interacting 1 pathways that might mediate this relationship (Ibáñez et al., 2014). In this review article, we will revisit the evidence for the association between SCZ and tumour suppressor genes. Efforts to understand this relationship may result in the identification of novel biomarkers for early SCZ diagnosis and aid in the exploration of SCZ pathology.

## The Correlation Between Tumour Suppressor Genes and SCZ

### Epidemiological Studies of Tumour Susceptibility in Patients With SCZ

The first argument for the relationship between SCZ and tumour suppressor genes is based on epidemiological surveys indicating reduced cancer risk in schizophrenics and their relatives. For example, a well-designed study found a significantly lower risk of cancer in patients with familial aggregated SCZ and their biological parents when compared to that in the general population (Gal et al., 2012). Similar results were obtained in sibling studies reporting a reduced cancer risk in the affected siblings of patients with SCZ (Levav et al., 2007). We speculate that the upregulation of tumour suppressor gene expression may confer susceptibility to SCZ. However, Lichtermann et al. (2001) provided contradictory evidence by showing that patients with SCZ had a higher risk of lung or pharyngeal cancer than their unaffected relatives, whose risk was lower than that in the general population. The authors argued that the elevated cancer risk in patients with SCZ was likely attributable to their unhealthy lifestyles, whereas the lower-than-normal risk in unaffected siblings might reflect the protective effects of increased tumour suppressor gene expression uncompromised by unhealthy lifestyle changes. In any case, this evidence is consistent with an interaction between SCZ and tumour suppressor gene function.

### Gene Association Studies Linking TP53 and Wnt Pathway Genes to SCZ

The earliest evidence correlating SCZ with a tumour suppressor gene was the finding that SCZ was associated with mutations in the *TP53* gene (Catts and Catts, 2000). This gene encodes the well-established tumour suppressor protein p53 (Jiang et al., 2011), and *TP53* mutations are frequently observed in various human cancers (Levine and Oren, 2009). The role of p53 in SCZ pathogenesis was further supported by three separate lines of evidence: (1) two new SCZ candidate genes were found on human chromosome 6q21, which was previously shown to contain a tumour suppressor gene (Morelli et al., 2000), and an SCZ-associated gene locus containing the common fragile site FRA6F was observed in various human leukaemias (Morelli et al., 2002); (2) increased apoptosis was reported to result in neurodevelopmental abnormalities, including SCZ (Sanders et al., 2013); and (3) p53 was reported to induce cellular apoptosis to prevent malignant transformation and tumour development (Vousden and Prives, 2009).

The involvement of multiple tumour suppressor genes in SCZ indicates that certain cellular mechanisms may regulate both tumourigenesis and neural function. Among such putative mechanisms, Wnt signalling is widely reported to be involved in SCZ pathogenesis (Peng et al., 2014). In fact, Wnt signalling is a pleiotropic pathway mediating nearly every aspect of cell growth, including tumorigenesis. For example, Wnt1 was identified as an oncogene (Nusse et al., 1984). It is not unexpected that the Wnt pathway can mediate SCZ by modulating neurodevelopment. In the canonical Wnt pathway, Akt kinase, which is a glycogen synthase kinase 3β inhibitor, and β-catenin are the major downstream effector proteins. An early study reported decreased β-catenin expression in the hippocampal regions of patients with SCZ (Cotter et al., 1998). More compelling evidence was recently obtained by demonstrating abnormal Wnt signalling in human-induced pluripotent stem cells from patients with SCZ during differentiation into neural progenitor cells (Topol et al., 2015). In addition, frizzled protein 7, which is a Wnt receptor, was recently found to be upregulated in patients with SCZ (Hoseth et al., 2018). The second tumour suppressor gene product of the canonical Wnt/β-catenin pathway to be associated with SCZ is adenomatous polyposis coli (APC). In an animal study using the N-methyl-D-aspartate receptor antagonist MK-801 to induce SCZ-like behaviours, *APC* gene expression in the prefrontal cortex and ventral tegmental area was associated with SCZ symptoms (Yu et al., 2011). Furthermore, a systematic study using the transmission disequilibrium test identified three single nucleotide polymorphisms (SNPs) of the *APC* gene that are correlated with SCZ (Cui et al., 2005). Taken together, these gene associations suggest a possible link between the Wnt signalling pathway and SCZ.

### Other Candidate Tumour Suppressor Genes Associated With SCZ

Other tumour suppressor genes have also been associated with SCZ. For example, transforming growth factor-beta type II serine/threonine kinase receptor on chromosome 3p22 was shown to be transcriptionally upregulated in patients with SCZ, and its transcription was normalised after antipsychotic treatment (Numata et al., 2008). Protocadherins have also been associated with SCZ and tumour suppressor functions (Kim et al., 2011). Similar to lung cancer, the prevalence of colorectal cancer is reported to be lower in SCZ cohorts than in unaffected individuals (Catts et al., 2008). In a detailed study, allele-specific expression of the mutated in colorectal cancer gene at the rs2227948 and rs2227947 loci was found to be significantly different between patients with SCZ and healthy individuals (Wang et al., 2016), suggesting that mutated in colorectal cancer, a potential tumour suppressor gene, might be involved in SCZ. The tumour suppressor gene histidine triad nucleotide-binding protein 1 is down-regulated in the prefrontal cortex of patients with SCZ (Elashoff et al., 2007). In human patients with SCZ, histidine triad nucleotide-binding protein 1 is associated with acute behavioural changes (Su et al., 2003), whereas histidine triad nucleotide-binding protein 1 knockout mice show elevated anxiety- and depression-like behaviours (Sun et al., 2017). We have summarised the major tumour suppressor genes associated with SCZ described to date in Table 1.

**Table 1:** Major tumour suppressor genes and loci contributing to schizophreni(SCZ) susceptibility.

Tumour suppressor gene	SCZ-related loci	Gene functions	References
*TP53* (p53)	rs2078486, D17S1566 marker	Suppressing tumourigenesis, promoting apoptosis	Catts and Catts (2000)
*WNT1*		Regulating cell growth	Peng et al. (2014) and Topol et al. (2015)
*CTNNB* (β-catenin)		Cell growth and metabolism	Cotter et al. (1998)
*FZD7*		Tissue development	Hoseth et al. (2018)
*APC*	rs2229992, rs42427, and rs465899	Regulation of cell proliferation and tissue development	Yu et al. (2011)
*PCDHs*		Suppressing tumour growth	Kim et al. (2011)
*MCC*		Suppression of colorectal cancer	Wang et al. (2016)
*HINT1*		Epigenetic regulation	Su et al. (2003)
*BRCA2, PALB2*	rs420256, rs9567552	Tumour suppressor	Tesli et al. (2010)
*BDNF*	rs6265	Neurotrophic factor	Zhang et al. (2018)

## Genetics of Tumour Suppressors and SCZ Susceptibility

Single gene mutations can present in multiple forms, from SNPs to whole-gene duplications or inversions/deletions (indels). Unique phenotypes are associated with different mutant alleles and loci. In genetic studies of SCZ, a number of different candidate loci have been identified. The relationship between p53 polymorphisms and SCZ was reported in an early study showing that patients carrying specific alleles had reduced vulnerability to lung cancer (Park et al., 2004). Specifically, whereas the rs1042522 locus (containing a Pro72Arg mutation and a 16-bp insertion) was not significantly associated with an increased risk of SCZ (Papiol et al., 2004), rs2078486 and other SNP loci in the *TP53* gene appeared to significantly increase or decrease SCZ susceptibility (Yang et al., 2004). In a subsequent study, transmission disequilibrium test-based analyses consistently linked specific p53 polymorphisms, including CAA indels and a 16-bp indel, to SCZ pathogenesis in independent patient cohorts (Ni et al., 2005). Moreover, a study of an isolated Spanish population revealed structural variations in *TP53* at the D17S1566 marker that conferred a high risk of SCZ (Tabarés-Seisdedos et al., 2008). A case-control study further elaborated the relationship between the *TP53* codon 72 polymorphism and SCZ risk (Lung et al., 2009), whereas *TP53* alleles carrying BstUI (exon 4) and MspI (intron 6) restriction sites were shown to confer greater susceptibility to lung cancer in Turkish patients with SCZ (Ozbey et al., 2011). Taken together, these studies demonstrate the existence of multiple *TP53* loci associated with increased susceptibility to SCZ.

Additional candidate genes and loci associated with SCZ continue to be identified. A genomic study focusing on human chromosome 8p has identified dozens of risk loci, encoding both protein and microRNA in each region, including various tumour suppressor genes with structural variations, such as copy number variants, microdeletions, and microduplications, all of which contribute to SCZ (Tabarés-Seisdedos and Rubenstein, 2009). The breast cancer 2 and partner and localiser of breast cancer 2 genes, two commonly known risk genes for breast cancer, have been reported to be associated with SCZ at the rs420256 and rs9567552 loci, respectively (Tesli et al., 2010). Polymorphisms of glutathione peroxidase, in which higher numbers of GAG repeats can increase the risk of SCZ, are associated with prostate and colon cancer development (Zmorzyński et al., 2015). The genome-wide association study approach has revealed other candidate loci conferring susceptibility to SCZ. The gene encoding the epigenetic reader bromodomain-containing protein 4 is a major risk locus in both breast cancer and SCZ (Zuber et al., 2017). A study of bromodomain-containing protein 4 expression revealed that the rs138880 allele was associated with SCZ (Dyrvig et al., 2017). Moreover, analysis of X-ray repair cross-complementing 4 polymorphisms revealed protective effects of the rs6452536 and rs35268 loci against SCZ and colorectal cancer in a Chinese population (Wang et al., 2010). A genome-wide association study of oral squamous cell carcinoma identified a novel SNP at the X-ray repair cross-complementing 4 rs1412115 locus, in which an A > G substitution increases the risk of oral squamous cell carcinoma and is also related to SCZ (Ma et al., 2015). Opposing evidence also exists, as another study of cervical squamous cell carcinoma revealed correlations between TATC or CAA indel polymorphisms and increased risk of both cancer and SCZ (Shi et al., 2012). Nonetheless, the convergence of genetic risk for multiple tumour types and SCZ strongly indicates common underlying cellular mechanisms. In examining the Wnt signalling pathway, the gene encoding a major Wnt receptor, frizzled-3, was found to have a number of SNPs associated with SCZ pathogenesis (Katsu et al., 2003). Similar patterns for SCZ-related SNPs have been found in a downstream Wnt effector gene, Dickkopf 4, in a Chinese population (Proitsi et al., 2008). In addition, three SNPs in *APC* exons that are associated with SCZ, including rs2229992, rs42427, and rs465899 have been identified (Chambers and Perrone-Bizzozero, 2004). The gene encoding brain-derived neurotropic factor, which is indirectly related to the Wnt pathway, has also been implicated in suppressing tumour pathogenesis (Cao et al., 2010), and its rs6265 SNP genotype has recently been found to affect SCZ pathogenesis (Zhang et al., 2018). The major tumour suppressor genes and loci related to SCZ are summarised in Table 1.

## Epigenetic Regulation of Tumour Suppressor Genes in SCZ

In addition to the direct regulation of protein expression and function by variants in protein-coding regions, epigenetic mechanisms play an important role in SCZ onset by regulating protein expression without altering the gene sequence. Evidence is continuously growing for the role of epigenetics in SCZ occurrence (Dempster et al., 2013). Mutations in both coding and non-coding regions can affect gene expression levels, thus affecting biological function. In a study investigating the correlation between SCZ risk and variants of the deleted in colorectal cancer gene, the SNP locus rs2270954 was found to reside in the 3’ untranslated region, thus presumably mediating gene function without affecting the protein sequence (Grant et al., 2012). Recent studies have shown that the correlation between SCZ and tumorigenesis may be regulated epigenetically through the involvement of microRNAs (Rizos et al., 2016). For example, miR-183, previously shown to mediate the expression of multiple tumour suppressor genes, was upregulated in a cohort of patients with SCZ (Rizos et al., 2012). Similarly, miR-137 may play a dual role in brain tumour suppression and neural development underlying psychiatric disease, including SCZ (Mahmoudi and Cairns, 2017). Another recently identified non-coding RNA, miR-193a-3p, is a circulatory marker for various tumours and has also been identified in patients with SCZ (Grossi et al., 2017). These studies have demonstrated that both genetic and epigenetic regulation of certain tumour suppressor genes contributes to the risk of SCZ.

## Cellular and Molecular Mechanisms of Tumour Suppressor Gene Involvement in SCZ

Although the abovementioned evidence highlights the correlation between tumour suppressor genes and SCZ susceptibility, we still lack evidence of a causal relationship. One possible hypothesis is that tumour suppressor genes participate in SCZ pathogenesis through neurodevelopment or neural functions. The first piece of supporting evidence for the above hypothesis was reported for the p53 gene, whose over-expression was shown to lead to excessive neuronal death and impaired neural function (Hughes et al., 1997). In patients with SCZ, p53 activation can enhance apoptosis in dermal fibroblasts (Catts et al., 2006) and stem-cell-derived fibroblasts from medication-free individuals (Gassó et al., 2014). Therefore, p53 hyper-activation may enhance tumour surveillance at the cost of higher neuronal apoptosis, which impairs psychiatric function. In addition, transcriptional regulation may help to explain the potentially causal relationship between tumour suppressor gene activity and SCZ. Dysbindin-1, the product of the SCZ risk gene dystrobrevin binding protein 1, increases *TP53* gene expression and leads to excessive neurite outgrowth (Ma et al., 2011). The above developmental effects of p53 highlight possible routes by which tumour suppressor- and SCZ-associated genes may interact to affect SCZ risk.

Other tumour suppressor genes may also contribute to SCZ pathogenesis due to their effects on neural development, including axon guidance and dendritic arborisation. They include SCZ risk genes such as disrupted in SCZ-1 (Miyoshi et al., 2003; Mackie et al., 2007). Wnt signalling is associated with SCZ pathogenesis and regulates brain development during both embryonic and postnatal stages (Inestrosa et al., 2012). Specifically, canonical and non-canonical Wnt pathways mediate axon guidance and dendritic arborisation, indicating possible roles in SCZ development. Aberrant Wnt signalling and decreased neurite numbers were observed in reprogrammed neurons derived from human-induced pluripotent stem cells in patients with SCZ (Brennand et al., 2011). Given that Wnt activation can facilitate tumour growth, its deactivating mutations may simultaneously reduce tumorigenesis and impair neuronal development, probably increasing SCZ risk. In addition to direct regulation of neural development, the Wnt pathway may be modified epigenetically, as the tumour-suppressing miR-137 can simultaneously modulate Akt pathway activity (Thomas et al., 2017) and the expression of brain-derived neurotrophic factor (Hill et al., 2014), highlighting its dual effects on tumour growth and SCZ risk. Downstream of the Wnt pathway, converging molecular pathways further orchestrate tumour cell surveillance and neurodevelopment. For example, heterozygous knockout of the tumour suppressor gene *APC* leads to development of age-dependent working memory deficits and hypoactivity (Koshimizu et al., 2011). A mutation in the mouse *APC* gene has also been reported to result in abnormal dendritic spine formation and long-term potentiation in hippocampal neurons, and is associated with impaired social interaction (Onouchi et al., 2014). Although some of the above findings are inconsistent with our proposed model, they still suggest the involvement of tumour-related genes in neural function.

SCZ pathogenesis is inherently a polygenic event involving interactions among multiple risk genes. A recent association study reported that the interaction between the SNP loci of two tumour suppressor genes, thioredoxin interacting protein and *AF1q*, contributed to SCZ susceptibility (Su et al., 2017). The molecular mechanisms underlying such gene interactions mainly resides in epigenetic or transcriptional regulation. For example, Wnt activation can epigenetically enhance the expression of transcription factor 4, which is a transcription factor for SCZ risk genes (Hennig et al., 2017). Interestingly, transcription factor 4 mediates apoptosis and epithelial-mesenchymal transition genes in addition to neurodevelopmental factors (Forrest et al., 2013), making it a potent “dual player” candidate for SCZ and cancer. Upstream of Wnt signalling, disrupted in SCZ-1 can stimulate the transcriptional activity of β-catenin (Boccitto et al., 2016) and upregulate Wnt activity. Outside the Wnt pathway, other tumour suppressor gene may also interact with SCZ risk genes, such as protein phosphatase 2A (Palanichamy et al., 2017).

In addition to converging molecular pathways and transcriptional regulation between tumour suppressor and SCZ risk genes, shared epigenetic regulation of these two groups of genes may confer SCZ and cancer phenotypes. For example, age-related DNA methylation can elevate the risk of both SCZ and cancer (Jenkins et al., 2014). Histone deacetylase 2 regulates metabotropic glutamate receptor 2 promoter activity (Kurita et al., 2012) in addition to p53 expression levels (Wagner et al., 2014). Therefore, epigenetic regulation of DNA represents another mechanism linking cancer risk and SCZ, although not all studies have reported results consistent with our hypothesis of an inverse correlation between cancer and SCZ risk. As an additional layer of gene function regulation, post-translational modification of tumour gene protein products has also been implicated in SCZ. One study reported that a novel tumour suppressor gene, zinc fingers DHHC-type 14, encoded an enzyme for S-acylation that is implicated in SCZ (Greaves and Chamberlain, 2014). Taken together, the separate lines of evidence discussed above support the existence of genetic and epigenetic regulatory networks underlying SCZ pathogenesis and tumorigenesis.

## Conclusion and Future Perspectives

**Figure 1:**
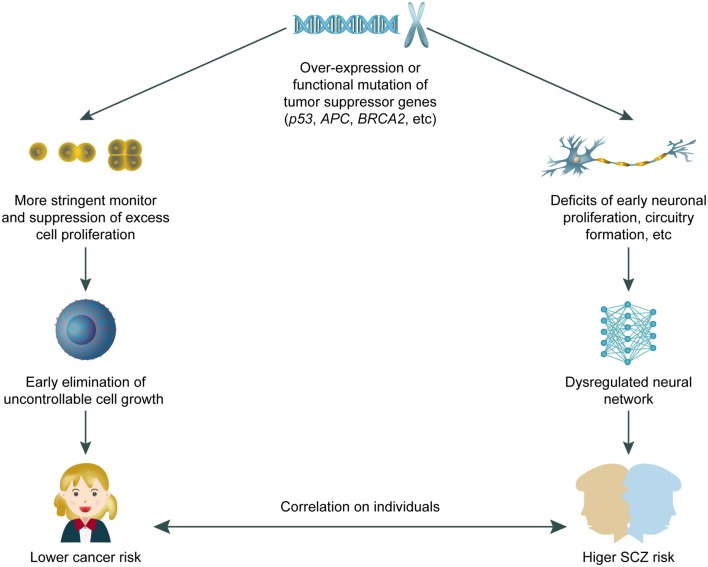
A working model for “double edged” effects of tumour suppressor genes on both cancer and schizophrenia (SCZ) risk. The over-expression or gain-of-function mutation of tumour suppressor genes confer dual effects including closer monitoring of unwanted cell proliferation, plus potential interruption on neural development that leads to higher SCZ risk in adults. The correlation and potentially “trade-off” between SCZ and cancer risk requires further study.

We have summarised the recent major progress in understanding the roles of tumour suppressor genes in SCZ pathogenesis and discussed potential underlying cellular and molecular mechanisms. Tumour suppressor genes have been associated with neurodevelopmental disorders other than SCZ, such as autism (Crespi, 2011). These findings reinforce the general concept that abnormalities in neurodevelopment can lead to a spectrum of behavioural deficits that spans autism, SCZ, and other syndromes. Research on the role of tumour suppressor genes in SCZ has important implications for drug development. Typical antipsychotics such as haloperidol can cause neuronal apoptosis via p53 activation, whilst atypical drugs can have the opposite effect of neuroprotection (Nandra and Agius, 2012). We therefore expect that atypical antipsychotic drugs should have better efficacy and fewer adverse effects in SCZ cases with tumour suppressor gene hyperactivation. In reviewing associations of SCZ with the Wnt pathway, we hypothesise that drugs targeting this pathway may be effective antipsychotics (Freyberg et al., 2010; Singh, 2013). The feasibility of correcting epigenetic abnormalities *in vivo* warrants more research and development as a potential pharmacological approach for SCZ. Histone deacetylase 2 is a critical mediator of atypical antipsychotic drug action (Kurita et al., 2012) and the tumorigenic activity of p53 (Wagner et al., 2014). While anti-tumour drugs targeting specific cellular and molecular pathways are being rapidly developed, antipsychotic drugs are meeting hurdles in the traditional path. Therefore, screening anti-tumour drugs for potential neural modulating properties may provide promising fast-track candidates for SCZ treatment. Conversely, recent studies have suggested that anti-psychotic drugs might be effective in decreasing cancer risk (Shi et al., 2015), which may partially explain the lower cancer incidence observed in patients with SCZ (Raviv et al., 2014; Xu et al., 2017). However, the anti-tumorigenic effect of anti-psychotic drugs is unlikely to fully explain those surveys. Rather, we hypothesise that tumour suppressor genes may protect patients with SCZ from cancer onset. This “double-edged sword” model (Figure 1) reflects the intricate balance between preventing dysregulated cell growth and protecting against abnormal neural development, in which a higher risk of tumour onset is traded for a lower incidence of SCZ, and vice versa. In summary, tumour suppressor genes and SCZ are highly correlated genetically, molecularly, and potentially pharmacologically. Further investigation is required to achieve a better, clinically translatable understanding of the cellular mechanisms of tumour suppressor gene involvement in neurodevelopment and behaviour.

## Hypothesis

Based on our current knowledge of tumour suppressor gene involvement in SCZ pathogenesis, we propose a working model in which at least certain SCZ cases can be attributed to functional hyperactivation of tumour suppressor genes, resulting in a concomitant reduction in susceptibility to cancers. Because tumour suppressor genes can inhibit cell proliferation and growth, they can also affect normal development or activity of neural tissues, thus leading to SCZ.

## Author Contributions

CZhuo: study idea and design. HT, SL, and JL: systematic review, extraction, and analysis. FJ, CC, CL, MC, CZhou, LZ, and CZhuo: drafting of the manuscript. CZhuo, XS, JL, DW, and LZ: critical revision of the manuscript for important intellectual content. CZhuo, CC, CL, JL, LZ, and DW: access to data and had full access to all the data in the study and take the responsibility for the integrity of the data and the accuracy of the data analysis. All authors have reviewed the manuscript.

## Conflict of Interest Statement

The authors declare that the research was conducted in the absence of any commercial or financial relationships that could be construed as a potential conflict of interest. The reviewer LC and the handling Editor declared their shared affiliation.
